# Polymer–Nematic Liquid Crystal Interface: On the Role of the Liquid Crystalline Molecular Structure and the Phase Sequence in Photoalignment

**DOI:** 10.3390/polym13020193

**Published:** 2021-01-07

**Authors:** Ameer R. K. Nassrah, István Jánossy, Viktor Kenderesi, Tibor Tóth-Katona

**Affiliations:** Wigner Research Centre for Physics, Institute for Solid State Physics and Optics, P.O. Box 49, H-1525 Budapest, Hungary; nassrah.ameer@wigner.hu (A.R.K.N.); janossy.istvan@wigner.hu (I.J.); kenderesi.viktor@wigner.hu (V.K.)

**Keywords:** nematic–polymer interface, photoalignment, optical sensors, optical actuators

## Abstract

We provide experimental evidence for the influence of the molecular structure of the nematic liquid crystal (NLC) on the photoalignment process in three dimensions at the interface with a polymer layer. In particular, the experimental findings are explained through the presence (or absence) of the π−π aromatic interactions between the NLC and the polymer. The influence of the nematic-to-smectic A phase transition on the photocontrol is also addressed. Furthermore, we demonstrate that the photo-induced reorientation scenarios can be eventually connected to conformational changes in the photosensitive polymer.

## 1. Introduction

The proper alignment of the molecules at the boundaries is the key factor for correct operation of devices based on liquid crystals (LCs). Standard, well established methods have been developed in the last decades to ensure the required orientation of liquid crystals [1] and have achieved high technological impact as being the basis of flat LC displays, the research and development of which have now moved mainly to industrial laboratories [2]. The interaction of liquid crystals with the bounding substrates, however, remained a particularly significant academic research area. Namely, there is a continuous search for alternative methods for aligning LCs that are applicable in diverse novel fields of LC research, such as micro-, nano-, and biotechnology, medicine, polymer and colloid science, photonics, etc. [3,4]. One of the most stimulating alternative method is the so-called photoalignment of nematic liquid crystals (NLCs), discovered almost three decades ago [5,6,7]. Photoalignment can be used not only to ensure the desired orientation in LC devices, but opens up the possibility to reorient (control) the liquid crystal director **n** through light irradiation in a contactless manner [8].

The photocontrol mechanisms, as well as the most common photosensitive materials are well described in a review on photoalignment of NLCs [9]. One of the most popular realization of the photoalignment is to exploit the trans-cis (E/Z) isomerization of azobenzene derivatives [10]. Two basic mechanisms of photoalignment are known in LC sandwich cells constructed with one photosensitive substrate (with either polymer-, or monolayer of an azobenzene derivative) and one traditionally prepared reference plate. In the first scenario the light irradiation creates cis (Z) isomers that trigger a homeotropic-to-planar transition—this is the so called ‘out-of-plane alignment’, or zenithal photocontrol [11,12,13]. In the other mechanism (‘in-plane alignment’, or azimuthal photocontrol), the liquid crystal molecules remain in the plane of the substrate and the azimuthal angle of the director is controlled with polarized light: after the irradiation the director becomes perpendicular to the light polarization direction [5,6,14,15].

Recent experiments on azimuthal photocontrol have shown, however, that the above classification of the photoalignment mechanisms is less unambiguous. Namely, beside the azimuthal photoalignment, in a certain temperature range, zenithal photoalignment has also been observed. Moreover, close to the nematic-to-isotropic phase transition of the LC interfacing the photosensitive layer, a temperature induced anchoring transition (from planar towards homeotropic) has also been detected. The results have been explained by the different temperature dependence of the azimuthal and zenithal anchoring strengths at the polymer-LC interface [16]. Furthermore, it has also been shown that the mechanism of the photoalignment depends on the liquid crystalline molecular structure: the photocontrol of LCs with biphenyl rigid cores substantially differs from that of LCs not having biphenyl group in the molecular structure. The differences have been phenomenologically explained by the presence/absence of the offset stacked π−π aromatic interactions between the rigid core of the LCs and the azobenzene moiety of the interfacing polymer [17].

In the current work we provide further evidences of our previous assumptions about the mechanism of photoalignment by using various new types of LCs in the experiments. In particular, we investigate a compound where one of the phenyl group has been modified with three fluorene atoms at 3,4,5 positions, which almost “invert” the molecular electrostatic surface potential (MESP) of the ring [18]. Additional test measurements have been also performed on a LC compound having similar molecular structure to the PCH homologous series investigated in our previous publication [17]. The third LC which we investigate is a cyanobiphenyl compound, exhibiting a nematic-to-smectic A phase transition at a temperature $T_{AN}$. This allows us to study the effect of pretransitional fluctuations on the photoalignment. Namely, both the bend and the twist elastic constants ($K_{33}$ and $K_{22}$, respectively) exhibit an increase (a divergence) near $T_{AN}$ due to pretransitional smectic fluctuations [19,20,21] which are expected to influence the photoalignment. Finally, we provide MESP for all investigated LC compounds, as well as some of the conformers of the photosensitive polymer, with the aid of which we discuss the possible photoalignment processes.

## 2. Materials and Methods

**Figure 1:**
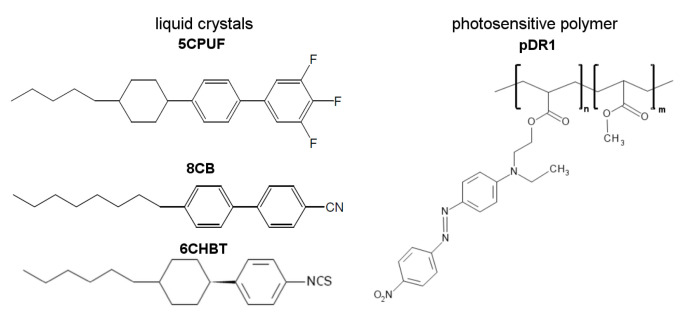
Molecular structure of liquid crystals 5CPUF, 8CB, 6CHBT and of the photosensitive polymer pDR1.

NLCs 3,4,5-trifluoro-4′-(4-pentyl-cyclohexyl)biphenyl (5CPUF) and 4-octyl-4′-cyanobi phenyl (8CB) have been purchased from Synthon Chemicals, while 1-(trans-4-hexylcyclohe xyl)-4-isothiocyanatobenzene (6CHBT) from Sigma-Aldrich and were used as received (Figure 1). The nematic-to-isotropic phase transition temperature $T_{NI}$ (clearing point) of 56.5 ${}^{{}^{\circ}}$C, 41.5 ${}^{{}^{\circ}}$C and 43.0 ${}^{{}^{\circ}}$C has been found for 5CPUF, 8CB and 6CHBT, respectively. Additionally, a smectic A-to-nematic phase transition has been detected at the temperature $T_{AN}$ = 33.0 ${}^{{}^{\circ}}$C for 8CB. The photosensitive polymer was polymethyl-methacrylate (PMMA) functionalized with the azo-dye Disperse Red 1 (pDR1—see Figure 1), obtained from Alphamicron Inc., Kent, OH, USA. The glass transition temperature of the pDR1 has been found around $T_{g}\approx 115$ ${}^{{}^{\circ}}$C, i.e., far above the clearing temperature $T_{NI}$ of the liquid crystals.

Sandwich cells of typical thickness d∼10 μm have been prepared: the NLCs have been enclosed between a reference and a photosensitive plate. The reference plates were rubbed polyimide slides from E.H.C. Co. (Japan), ensuring a fixed, planar orientation of NLC (the director **n** parallel with the surface of the plate) at the surface. The photosensitive plates have been prepared by spin-coating pDR1 on the glass substrate as described in details in Ref. [16]. The pDR1 polymer has been dissolved in toluene in concentration of 2 wt.%. Spin-coating has been performed at 800 rpm for 5 s, and then at 3000 rpm for 30 s (all with spin acceleration of ±1000 rpm/s). The spin-coated substrates have been annealed preferably overnight in an oven (or alternatively, at least for 2 h) at 140 ${}^{{}^{\circ}}$C. The thickness of the pDR1 layer has been estimated to be in the order of ∼0.1 μm, based on the spin-coating experiments on PMMA [22]. The two plates have been assembled with spacers, and the thickness of the assembled cells have been measured by interferometric method. Liquid crystal materials have been filled in the cells in the nematic phase, few ${}^{{}^{\circ}}$C below the clearing temperatures, $T_{NI}$. Prior and during filling the cell with the NLC, the cell was illuminated with light polarized perpendicular to the rubbing direction on the reference plate, resulting in a good quality planar initial orientation of the NLC at both bounding substrates.

**Figure 2:**
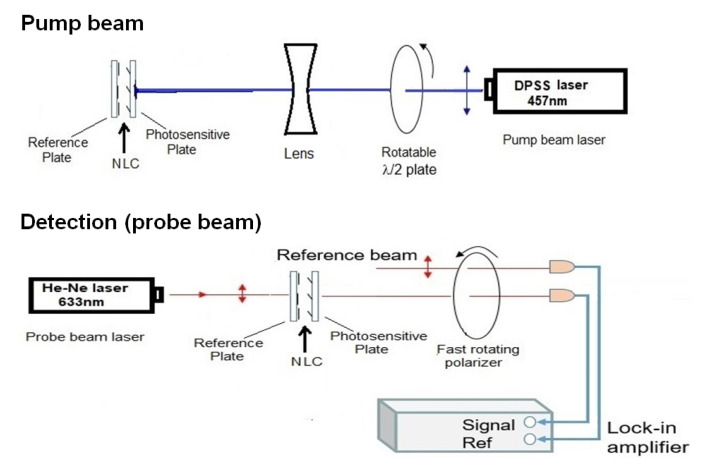
Experimental setup based on pump-probe beam technique, combined with a lock-in amplifier.

The experimental setup (Figure 2) for photoalignment measurements is similar to that presented in Ref. [23]. It is a pump-probe optical setup combined with a lock-in amplifier. The pump beam of a DPSS laser (≈20 mW, 457 nm) entered the NLC cell from the photosensitive side. The polarization direction of the pump beam has been regulated by a rotatable λ/2 plate, and the diameter of the beam has been expanded by a lens to a few mm (much larger than the diameter of the probe beam). The probe beam (He-Ne laser, 5 mW, 633 nm) entered the NLC cell at the reference plate and behind the sample it was sent through a rotating polarizer. The intensity of the probe-, and of the reference beam has been detected with photodiodes and the signals were connected to a lock-in amplifier (see Figure 2). The lock-in amplifier provides the phase and the amplitude of the probe beam.

In order to study the *in-plane* component of photoalignment, the polarization direction of the pump beam has been set parallel with the initial director orientation **n**. For the determination of the azimuthal photoalignment angle, φ, the polarization direction of the probe beam (and that of the reference beam) has been set parallel with the rubbing direction on the reference plate. The polarization direction of the transmitted probe, which is parallel to the director on the photosensitive plate, has been deducted from the phase of the lock-in signal (see Figure 2). $\varphi =90^{{}^{\circ}}$ corresponds to a complete azimuthal reorientation, i.e., the orientation on the photosensitive plate is perpendicular to that on the reference plate. The pump beam has been switched on at a certain moment (when the phase without the pump beam is determined), and is switched off after the saturation of the signal (reaching the value of $\varphi_{sat}$, typically after 3 min) enabling to follow the back-relaxation.

To detect the *zenithal photoalignment*, the polarization direction of pump beam has been set perpendicular to **n** (i.e., no azimuthal photoalignment is expected), while the probe beam was polarized at $45^{{}^{\circ}}$ from **n**, and the amplitude of the signal was measured. With this arrangement, if a significant out-of-plane photoalignment occurs, oscillations in the transmitted light intensity of the probe beam should appear, similarly to the measurements on the electric-, or magnetic-field induced Fréedericksz transition—see e.g., Ref. [24]. We note, that for a quantitative estimate of the zenithal photoalignment angle, $\theta_{photo}$, besides the sample thickness *d*, the temperature dependence of all relevant material parameters have to be known for the NLC.

For the calculation of the molecular electrostatic surface potential (MESP) of the NLC molecules and the conformational changes in polymer pDR1 the MarvinSketch program has been used.

## 3. Results

### 3.1. Azimuthal Photoalignment (In-Plane Photocontrol)

**Figure 3:**
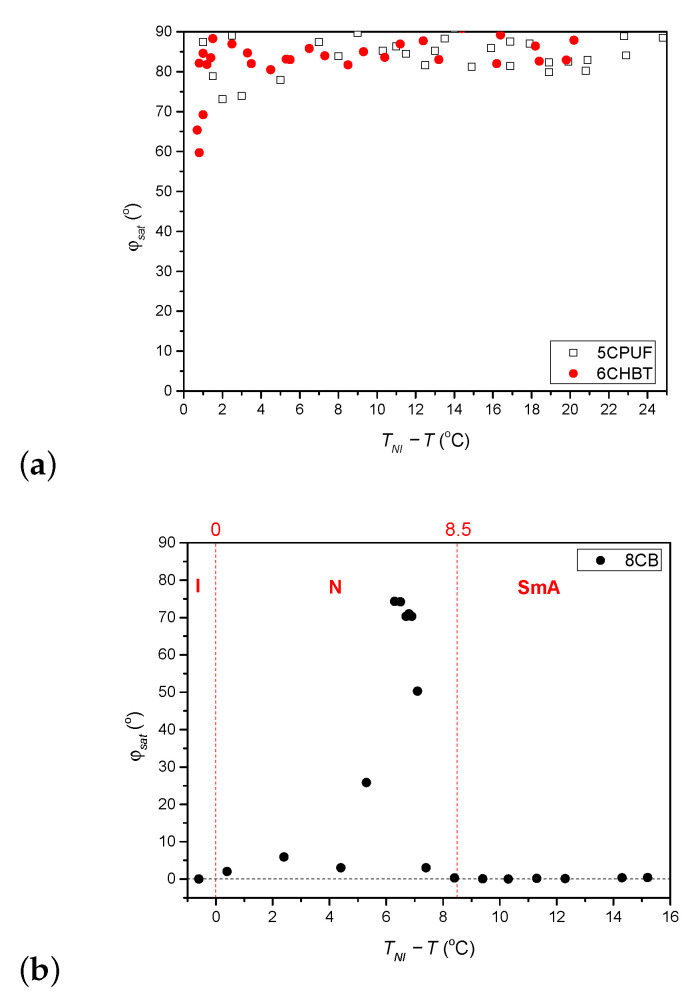
Temperature dependence of the saturated azimuthal photoalignment angle $\varphi_{sat}$ measured for (**a**) 5CPUF and 6CHBT, and (**b**) 8CB liquid crystals (I, N and SmA indicate the temperature range of isotropic, nematic and smectic A phase, respectively).

The temperature dependence (relative to the nematic-to-isotropic phase transition temperature, $T_{NI}$) of the saturated azimuthal photoalignment angle, $\varphi_{sat}$ measured for 6CHBT and 5CPUF is given in Figure 3a, while for 8CB in Figure 3b. In Figure 3b, the temperature ranges of the isotropic (I), nematic (N) and smectic A (SmA) phases are also indicated with the nematic-to-smectic A phase transition temperature at $T_{NI}-T=8.5$ ${}^{{}^{\circ}}$C.

**Figure 4:**
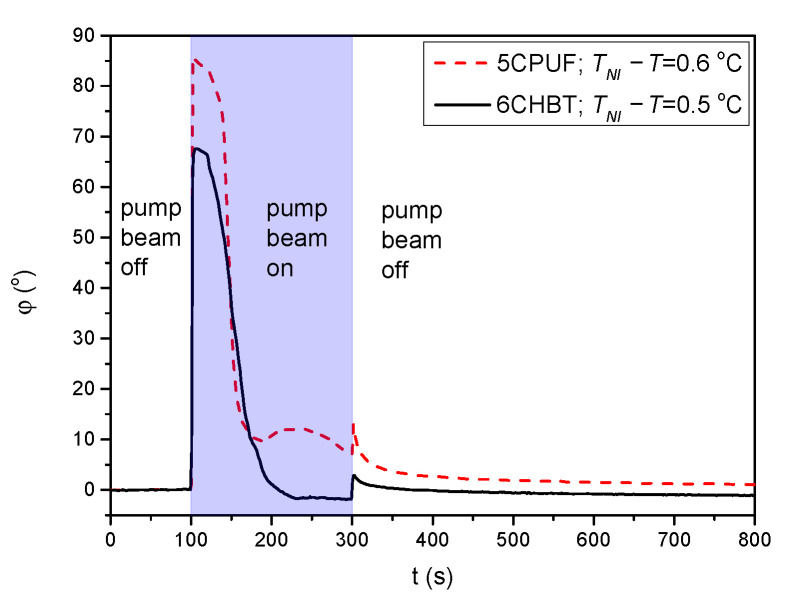
Temporal evolution of the azimuthal photoalignment angle φ measured for 5CPUF and 6CHBT at temperatures close to the phase transition temperature $T_{NI}$.

For 6CHBT and 5CPUF a nearly complete azimuthal photoalignment has been detected in the whole temperature range of the nematic phase, with $\varphi_{sat}$ typically in the range between $80^{{}^{\circ}}$ to $90^{{}^{\circ}}$. Close to the nematic-to-isotropic phase transition ($T_{NI}-T\leq 2$ ${}^{{}^{\circ}}$C) a slight decrease of $\varphi_{sat}$ has been observed, especially in the case of 6CHBT—see Figure 3a. Even closer to $T_{NI}$, at $T_{NI}-T<1$ ${}^{{}^{\circ}}$C the measurements on the photoalignment angle have failed, because of the thermal effect of the pump beam. This is illustrated with temporal evolution of the signal in Figure 4 for both 6CHBT and 5CPUF: when the pump beam is switched on (at t=100 s) the photoalignment immediately takes place, however, before it ends, the absorbed light heats up the NLC layer at the photosensitive substrate and it undergoes the transition into the isotropic phase within seconds and the photoalignment angle becomes meaningless. Similarly, when the pump beam is switched off, the nematic phase reappears within seconds and the signal tends towards the initial value.

The temperature dependence of the saturated azimuthal photoalignment angle, $\varphi_{sat}$, for 8CB (Figure 3b) has been found different from those obtained for 6CHBT and 5CPUF (Figure 3a). At low temperatures, in the smectic A (SmA) phase ($T_{NI}-T>8.5$ ${}^{{}^{\circ}}$C) no azimuthal photoalignment has been observed ($\varphi_{sat}=0$). In the nematic (N) phase, at temperatures close to the SmA phase (7.1 ${}^{{}^{\circ}}C<T_{NI}-T<8.5$ ${}^{{}^{\circ}}C$) no significant azimuthal photoalignment has been detected ($\varphi_{sat}<5^{{}^{\circ}}$). With the further increase of the temperature in a narrow range of 5.3 ${}^{{}^{\circ}}C\leq T_{NI}-T\leq 7.1$ ${}^{{}^{\circ}}C$ an incomplete azimuthal photoalignment has been measured ($25^{{}^{\circ}}<\varphi_{sat}<75^{{}^{\circ}}$). In the high temperature range of the nematic phase $0<T_{NI}-T<5.3$ ${}^{{}^{\circ}}C$ a negligible azimuthal photoalignment has been found again ($\varphi_{sat}<6^{{}^{\circ}}$), while the definition of $\varphi_{sat}$ in the I phase ($T_{NI}-T<0$) becomes meaningless.

### 3.2. Zenithal Photoalignment (Out-Of-Plane Photocontrol)

**Figure 5:**
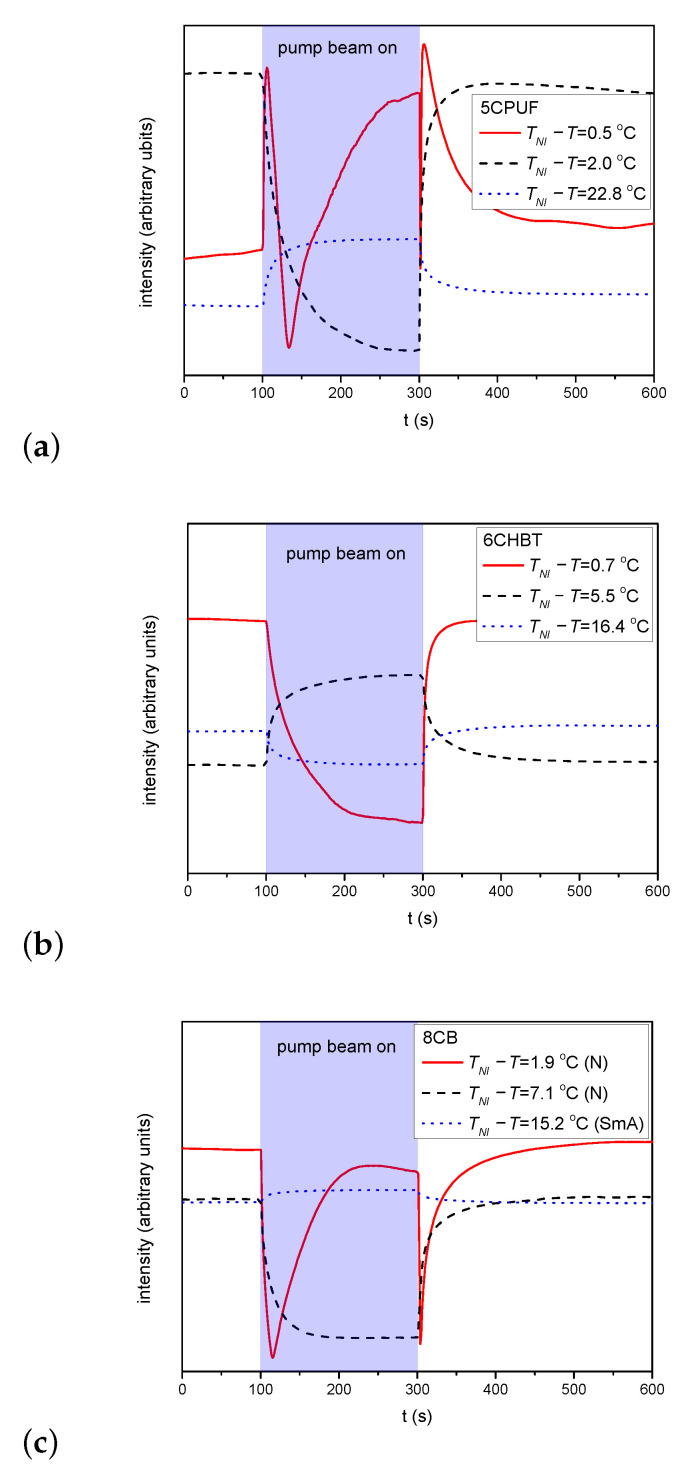
Temporal variation of the transmitted light intensity of the probe beam measured in cells with (**a**) 5CPUF, (**b**) 6CHBT, (**c**) 8CB at different temperatures, in the setup for detection of zenithal photoreorientation (pump beam polarization direction perpendicular to **n**, probe beam polarization direction encloses 45° with **n**).

For the estimation of the zenithal photoalignment the temporal evolution of the transmitted light intensity has been monitored in the experimental setup as described above in Section 2. Figure 5a–c shows the time evolution of the transmitted light intensity measured at different temperatures $T_{NI}-T$ for NLCs 5CPUF, 6CHBT and 8CB, respectively. The pump beam has been switched on at t=100 s and off at t=300 s in all cases as indicated in Figure 5.

For 5CPUF no oscillations in the transmitted light intensity is detected at any temperature when the pump beam is turned on/off, except very close to the clearing point—see at $T_{NI}-T=0.5$ ${}^{{}^{\circ}}$C in Figure 5a. These oscillations, however, appear not because of a significant zenithal photoalignment, but because of the thermal effect of the pump beam: the pump beam heats the sample and drives it through a nematic-to-isotropic phase transition as illustrated in Figure 4.

Similarly to 5CPUF, in samples with 6CHBT no oscillations in the transmitted light intensity of the probe beam have been observed either—see Figure 5b.

In the SmA phase of 8CB, the pump beam leaves almost unchanged the transmitted intensity of the probe beam—see Figure 5c for $T_{NI}-T=15.2$ ${}^{{}^{\circ}}$C. In the low temperature range of the nematic phase (5.3 ${}^{{}^{\circ}}C\leq T_{NI}-T<8.5$ ${}^{{}^{\circ}}C$) the transmitted light intensity change increases upon the application of the pump beam (see Figure 5c for $T_{NI}-T=7.1$ ${}^{{}^{\circ}}$C), however, temporal oscillations in the intensity have not been observed. On the other hand, in the high temperature range of the N phase (0 < $T_{NI}-T<5.3$ ${}^{{}^{\circ}}C$), where no significant azimuthal photoalignment has been observed, the transmitted light intensity oscillates when the pump beam is switched on/off (see Figure 5c for $T_{NI}-T=1.9$ ${}^{{}^{\circ}}$C) indicating a considerable zenithal photoalignment. Namely, in contrast to 5CPUF, the oscillations have been observed far below $T_{NI}$, thus can not be the consequence of the nematic-to-isotropic phase transition due to the heating effect of the pump beam.

### 3.3. Back-Relaxation

**Figure 6:**
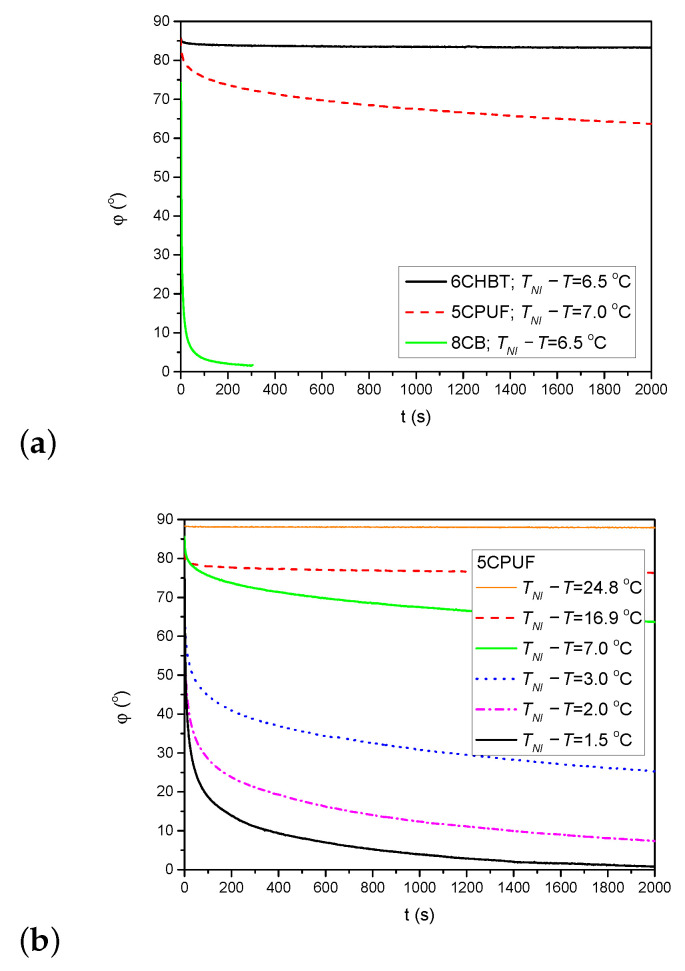
(**a**) Back-relaxation of the azimuthal photoalignment angle, φ for 6CHBT, 5CPUF and 8CB at relative temperatures $T_{NI}-T$ indicated in the legend. (**b**) Temperature dependence of the back-relaxation dynamics in the 5CPUF NLC.

The back-relaxation of the azimuthal photoalignment when the pump beam is switched off has also been monitored through the measurements on the time evolution of the azimuthal photoalignment angle φ. Figure 6a shows the time dependence of φ after the pump beam is switched off (at t=0 s) for 6CHBT, 5CPUF and 8CB at nearly the same temperature $T_{NI}-T$ as indicated in the legend. Obviously, the back-relaxation of 8CB is by orders of magnitude faster than in 5CPUF and in 6CHBT, and the relaxation in 5CPUF is somewhat faster than in 6CHBT.

Considering the temperature dependence of the back-relaxation dynamics, the general rule for all NLCs (5CPUF, 6CHBT and 8CB) is that it becomes faster with the increase of the temperature (i.e., with the decrease of $T_{NI}-T$), as it has also been found for other NLCs previously (for PCH homologous series, E7, E63, and ZLI1695) [16,17]. Here, we illustrate such a temperature dependence for 5CPUF in Figure 6b.

### 3.4. Molecular Electrostatic Surface Potentials (Mesps) and Conformations

**Figure 7:**
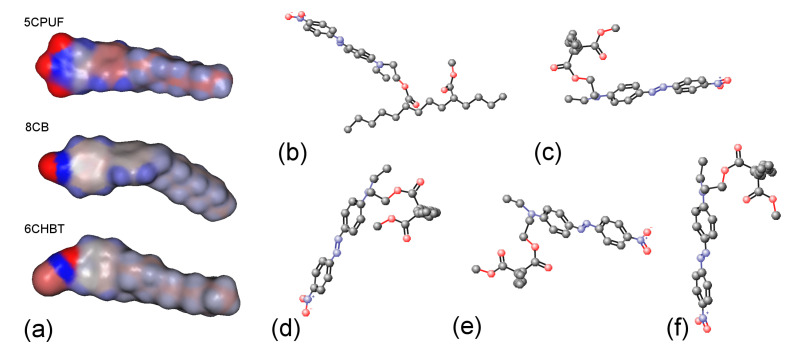
(**a**) Molecular electrostatic potentials (MESPs, blue is positive, red is negative) mapped for 5CPUF, 8CB and 6CHBT molecules; (**b**–**f**) some examples of various conformers of a pDR1 segment.

Molecular electrostatic surface potentials (MESPs) of the NLCs 5CPUF, 8CB and 6CHBT are presented in Figure 7a with a colour code blue positive, red negative. As expected [18], the three fluorine atoms in the structure of 5CPUF change the MESP of the phenyl ring considerably compared to the cyano-group in 8CB. In contrast to that, the thiocyanate (SCN) group in 6CHBT does not change significantly the MESP of the phenyl ring compared to the cyano-group in 8CB. Therefore, in this respect, 6CHBT can be regarded very similar to the benzonitrile containing PCH LC homologous series discussed very recently [17].

Possible conformations of the photosenitive polymer, pDR1, have been also investigated. For simplicity, only a segment shown in Figure 7b has been considered. Even for such an oversimplified polymer segment about 100 different conformations have been found, with most of them having energy difference in the order of RT=0.593 kcal/mol. We illustrate some of these conformers in Figure 7b–f, having energies of 127.74 kcal/mol, 126.62 kcal/mol, 125.96 kcal/mol, 134.14 kcal/mol, and 126.10 kcal/mol, respectively. For further simplification, in Figure 7c–f we have fixed the segment of the main-chain (visible in Figure 7b) perpendicular to the plain of the pictures. Such a representation clearly shows that in principle the trans-isomer of the azobenzene moiety can take any direction (from horizontal to vertical) at an energy expense of few RT, more likely due to the flexibility of the main chain than to the flexibility of the short spacer that connects the azo-dye with the polymer chain.

## 4. Discussion

The choice of the NLC compounds has been motivated by the main goals of the present work, namely:

- to confirm the role of the presence/absence of the offset stacked aromatic π−π interactions between the NLC and the photosensitive polymer, postulated recently [17];
- to get further insights in the role of the molecular structure of the NLC in photoalignment processes;
- to find out how the pretransitional fluctuations near the nematic-to-smectic A phase transition temperature influence the photoalignment.

To achieve these goals, both the azimuthal and zenithal photoalignment process, as well as the back-relaxation have been monitored. Additionally, the MESP of the NLCs have been constructed, and possible conformational changes in the photosensitive polymer have been considered.

**Figure 8:**
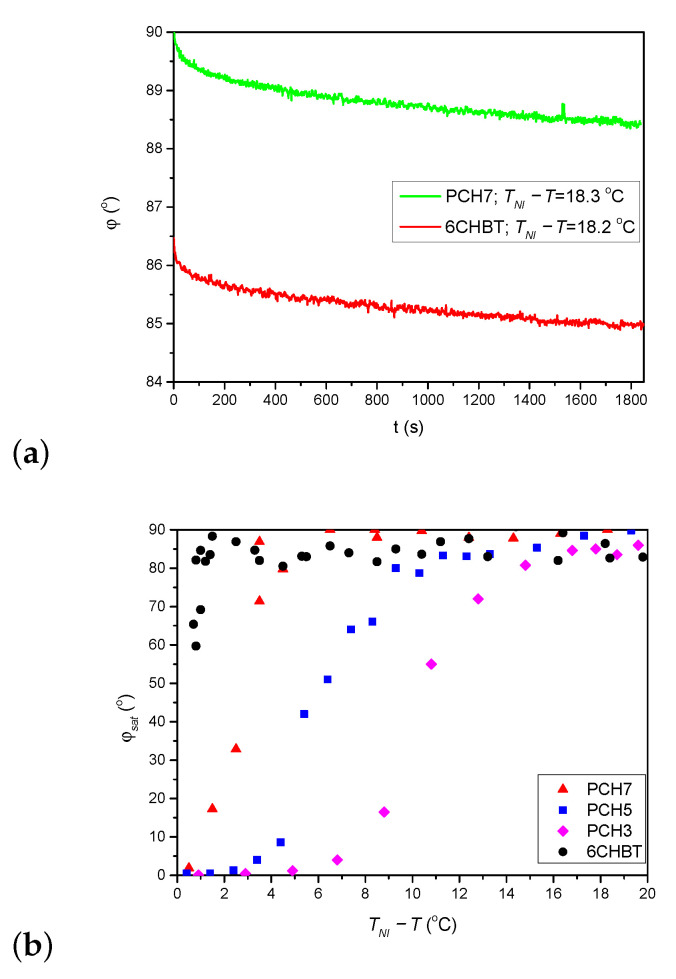
(**a**) Comparison of the back-relaxation of the azimuthal photoalignment angle, φ for 6CHBT and PCH7 at a relative temperature $T_{NI}-T\approx 18$ ${}^{{}^{\circ}}$C as indicated in the legend (data for PCH7 taken from [17]). (**b**) Temperature dependence of the saturated azimuthal photoalignment angle, $\varphi_{sat}$ for 6CHBT and for the PCH homologous series (data for PCH NLCs taken from [17]).

As stated above, the thiocyanate (SCN) group in 6CHBT does not change significantly the MESP of the adjacent phenyl ring compared to the cyano-group (CN) in 8CB (see Figure 7a). On the other hand, 6CHBT has similar molecular structure with phenylcyclohexane rigid core as PCH homologue series investigated recently [17], except the phenyl ring is connected to the SCN polar group instead of CN group in PCH LCs. Therefore, the photoalignment at the pDR1–6CHBT interface is expected to have the same characteristics as those at the interface pDR1–PCH homologue series, namely, efficient azimuthal photoalignment over a wide temperature range of the nematic phase and no considerable zenithal photoalignment [17]. This expectation is now confirmed by the experimental results: a complete (or close to complete) azimuthal photoalignment has been found almost in the whole temperature range of the nematic phase (except very close to $T_{NI}$—see Figure 3a), and no considerable zenithal photoalignment is detected (Figure 5b), similarly to the results obtained for the PCH homologous series [17]. Moreover, the back-relaxation dynamics of 6CHBT resembles to those obtained for the PCH NLCs. This fact is illustrated in Figure 8a, where the relaxation in 6CHBT is compared with that in PCH7 at about the same relative temperature $T_{NI}-T\approx 18^{{}^{\circ}}$C.

In Ref. [17] the question of possible role of the length of the alkyl chain in photoalignment has been also raised. It was found that the temperature difference $T_{NI}-T$, at which the saturation angle $\varphi_{sat}$ sharply drops, decreases as the length of the alkyl chain increases (see in Figure 8b). Considering that 6CHBT in other aspects of photoalignment shares the characteristics with the PCH homologous series, it is straightforward to make a comparison in this respect too. The comparison is shown in Figure 8b. Clearly, the azimuthal photoalignment is maintained in 6CHBT the closest to $T_{NI}$, despite it has a shorter alkyl chain than PCH7 (and has the lowest $T_{NI}$ among these NLCs on the absolute scale). Therefore, besides the role of the length of the alkyl chain (if any), some other factor(s) influences the process too, which will be considered in the future.

In the case of 5CPUF, the three fluorine atoms attached to the phenyl ring in the structure change the MESP of the adjacent ring (and even that of the next phenyl ring) to a large extent compared to that of the cyanobiphenyl 8CB (see Figure 7a). These modifications in the MESP of the biphenyl part of the 5CPUF molecules prevent the offset stacked π−π aromatic interactions between the biphenyl part of the 5CPUF and the azobenzene moiety of the interfacing polymer as it has been outlined recently—see Figure 9 of [17], and the corresponding explanation there. Therefore, the same characteristics of the photoalignment are expected for 5CPUF as those found in NLCs with phenylcyclohexane, or bicyclohexane rigid core (6CHBT described here, PCH homologous series and ZLI1695 described in [17]), namely, (i.) a complete (or nearly complete) azimuthal photoalignment in the whole temperature range of the nematic phase (except very close to $T_{NI}$, where thermal effect of the pump beam, or the eventual temperature induced anchoring transition [16] may have influence); (ii.) no considerable zenithal photoalignment; (iii.) in the absence of the offset stacked π−π aromatic interactions between 5CPUF and the polymer, a much slower back-relaxation than in the cyanobiphenyl NLCs. The experimental results on 5CPUF presented here underpin all these expectations: a (nearly) complete azimuthal photoalignment has been found in the whole temperature range of the nematic phase (see Figure 3a), no considerable zenithal photoalignment detected (see Figure 5a), and the back-relaxation is much slower in 5CPUF than in 8CB (see Figure 6a), or in other cyanobiphenyl NLCs, like 5CB, E7, and E63 (see in [16]).

Photoalignment of cyanobiphenyl NLC compounds (5CB, mixtures E7 and E63) interfacing the pDR1 polymer has been extensively studied recently [16,17]). In these systems an incomplete azimuthal photoalignment has been measured over a wide temperature range of the nematic phase, while a considerable zenithal photoalignment has been found in the temperature range in which the azimuthal photoalignment is inefficient. Furthermore, just below $T_{NI}$, a temperature induced anchoring transition has been detected from planar towards homeotropic orientation of cyanobiphenyl NLCs E7 and 5CB. Moreover, the back-relaxation of the cyanobiphenyl compounds from the photo-reoriented state has been found much faster than in NLCs with phenylcyclohexane, or bicyclohexane rigid core. These experimental results have been explained by the different temperature dependencies of the azimuthal and zenithal anchoring strengths at the NLC–polymer interface [16], and by the offset stacked π−π interactions between the biphenyl core of the NLCs and the azobenzene moiety of the polymer that induce additional stresses acting against the photoinduced *trans-cis* isomerization in the system [17].

In the nematic phase, the 8CB cyanobiphenyl compound is expected to possess similar photoalignment characteristics as the above mentioned cyanobiphenyl NLC compounds, except slightly above the nematic-to-smectic A phase transition temperature, $T_{AN}$, where pretransitional smectic fluctuations are present. The experiments reported here confirm most of these expectations, namely, in the high temperature range of the nematic phase ($0<T_{NI}-T<5.3$ ${}^{{}^{\circ}}$C) no considerable azimuthal photoalignment has been detected (see Figure 3b), while the oscillation in the amplitude of the transmitted light intensity indicates considerable zenithal photoalignment (see Figure 5c at $T_{NI}-T=1.9$ ${}^{{}^{\circ}}$C). With the decrease of the temperature, in the narrow range of 5.3 ${}^{{}^{\circ}}$C $\leq T_{NI}-T\leq 7.1$ ${}^{{}^{\circ}}$C an incomplete azimuthal photoalignment (Figure 3b), and no considerable zenithal photoalignment (see Figure 5c at $T_{NI}-T=7.1$ ${}^{{}^{\circ}}$C) has been found. With further decrease of the temperature, in the nematic phase (7.1 ${}^{{}^{\circ}}$C $<T_{NI}-T<8.5$ ${}^{{}^{\circ}}$C), as well as in the smectic A phase ($T_{NI}-T>8.5$ ${}^{{}^{\circ}}$C), both the azimuthal and the zenithal photoalignment vanish. In the nematic phase it is presumably due to the pretransitional fluctuations, which significantly increase the bend and twist elastic constants [19,20,21], that are the most important parameters for the azimuthal and zenithal photoalignment, respectively. In the smectic A phase no azimuthal, nor zenithal photoalignment is expected, since there, the bend and twist deformations involve changes in the layer spacing and so are likely to be of very high energy [25].

One should note here, that 5CB and 8CB are compounds of the same homologous series (8CB having a longer alkyl chain), and that the temperature range of the nematic phase (from the room temperature) is roughly the same. In 5CB no considerable azimuthal photoalignment has been observed down to $T_{NI}-T\approx 10$ ${}^{{}^{\circ}}$C (room temperature—see Figure 2 of [16]). In contrast, in 8CB a relatively large (though incomplete) azimuthal reorientation has been found already at $T_{NI}-T\approx 5.3$ ${}^{{}^{\circ}}$C—see Figure 3b. Consequently, the question of possible role of the length of the alkyl chain in photoalignment mentioned above (and discussed for PCH series in [17]) rises again. However, the absolute value of $T_{NI}$ is higher for 8CB than for 5CB, similarly to the case for PCH compounds, where $T_{NI}$ increases with the increase of the length of the alkyl chain. Therefore, the clarification of this question remains for future studies.

Finally, we discuss conformations of the photosensitive polymer, pDR1. We are aware that considerations made in Section 3.4, and partially shown in Figure 7b–f are oversimplified: they consider only a particular (one of the most simple) possible segment of the polymer chain, do not take into account the whole polymer chain, the entanglements and the interactions between the chains, the actual phase of the polymer, etc. However, to our belief, even such an oversimplified picture can give some hints that may bring us closer to understanding the photoalignment process at the pDR1–NLC interface. Namely, for the explanation of experimental results obtained in [16] (including the temperature induced anchoring transition), a different temperature dependence of the zenithal and azimuthal anchoring strengths at the pDR1–NLC interface has been proposed. The different temperature dependencies of the anchoring strengths have been associated with the flexibility of the spacer consisting of two methylene units connecting the PMMA main chain with the azobenzene moiety (see pDR1 in Figure 1) [17].

Analysing the conformers of the pDR1 segment, and observing the Figure 7c–f, however, it seems more likely, that for the different temperature dependence of the zenithal and azimuthal anchoring strengths the flexibility of the PMMA polymer main chain is responsible (rather than that of the two methylene units spacer). As one can see in Figure 7c–f, the trans-isomer of the azobenzene moiety can take almost any direction (from horizontal to vertical) at an energy expense of few RT. The above assumption is supported by our latest, preliminary experiments at the interface of the derivatized methyl red (dMR) monolayer and cyanobiphenyl NLCs, 5CB as well as mixture E7 [26]. The dMR monolayer has been prepared by chemisorption of its triethoxysilane unit on the activated glass substrate. The triethoxysilane unit is connected to the azobenzene containing methyl red via three methylene units, i.e., the spacer unit here is longer than in pDR1. For the molecular structure of dMR, and for the preparation procedure see, e.g, [27]. Our preliminary measurements on these systems have shown a quite efficient azimuthal photoalignment of 5CB and E7 over the whole temperature range of the nematic phase, an absence of zenithal photoalignment, and a much slower back-relaxation than in systems with pDR1. All these observations indicate that the eventual flexibility of the methylene spacer does not play a role in the three-dimensional photoalignment, seen at the interface of cyanobiphenyl NLCs with pDR1.

## 5. Conclusions

In conclusion, the present paper contributes to a better understanding of the photoalignment process in systems in which the pDR1 polymer interfaces NLCs of various molecular structures. Furthermore, it tackles the influence of the pretransitional smectic fluctuations on the photoalignment. In particular, we have shown:

- Replacement of the cyanic polar head with fluorine atoms in NLCs having biphenyl in the rigid core (like in 5CPUF) modifies the MESP of the phenyl ring(s) to a large extent. This modification prevents the offset stacked π−π aromatic interactions between the biphenyl part of NLC and the azobenzene moiety of the interfacing polymer, and changes the characteristics of the photoalignment: instead of three-dimensional photoalignment (like in 8CB, as well as in 5CB, E7 and E63 [16,17]), the photoalignment occurs in two-dimensions (in-plane) only.
- Pretransitional smectic fluctuations suppress the photoalignment (both azimuthal and zenithal) in the nematic phase, presumably due to the large increase of the bend and twist elastic constants.
- 6CHBT NLC compound, having similar molecular structure to the PCH homologous series, exhibits similar photoalignment characteristics as those for PCH NLCs.
- From the analysis of the possible conformations on the simplest segment of pDR1, and from the preliminary measurements on dMR monolayer [26], it is more likely that the flexibility of the PMMA polymer main chain is responsible for the different temperature dependence of the zenithal and azimuthal anchoring strengths at the pDR1–NLC interface, and not the flexibility of the two methylene units spacer (as it has been proposed in [17]).
- Comparing the results on 8CB and 5CB (from [16]) shows that the efficiency of azimuthal photoalignment is maintained to temperatures closer to $T_{NI}$ as the length of the alkyl chain is increased. However, the role of the length of the alkyl chain is to be clarified in future studies.

## Data Availability

The data presented in this study are available on request from the corresponding author.
